# How the vertebrates were made: selective pruning of a double-duplicated genome

**DOI:** 10.1186/1741-7007-8-144

**Published:** 2010-12-13

**Authors:** Gerard Manning, Eric Scheeff

**Affiliations:** 1 Razavi Newman Center for Bioinformatics, Salk Institute for Biological Studies, 10010 North Torrey Pines Road, La Jolla, CA 92037, USA

## Abstract

Vertebrates are the result of an ancient double duplication of the genome. A new study published in *BMC Biology *explores the selective retention of genes after this event, finding an extensive enrichment of signaling proteins and transcription factors. Analysis of their expression patterns, interactions and subsequent history reflect the forces that drove their evolution, and with it the evolution of vertebrate complexity.

See research article: http://www.biomedcentral.com/1741-7007/8/146/abstract

## Commentary

A doubling of the genome, or whole genome duplication (WGD), is usually a cataclysmic event for an organism. Yet this polyploidy has been an important, if rare, event in the evolution of many plant groups, and has also occurred in yeasts, ciliates, fish and frogs [1]. It is now generally accepted that we and all other jawed vertebrates are the product of a remarkable two rounds of WGD, known as 2R [2], which duplicated every gene up to four-fold (fish and frog genomes have undergone a third duplication more recently). This opened the door to a tremendous expansion in functionality, and while most WGD duplicates, or ohnologs, were rapidly lost, this phenomenon was the genesis of almost one-third of all human genes. Establishing why these duplicates were retained and how they have evolved since then is an important way to advance the understanding of their current functions.

A study by Huminiecki and Heldin in *BMC Biology *[3] seeks to answer these questions through a global analysis of genes that survived the massive pruning that followed 2R. They identified 2R-derived gene pairs using a combination of sequence similarity (by comparing gene trees with the underlying species trees to identify duplications [4]) and chromosomal location, using syntenic chromosomal regions, in which runs of related gene pairs occur in different loci. They then explore the history of most vertebrate genes through 2R and subsequent gains and losses. They find that retained ohnologs are highly biased towards signaling genes and transcription factors and argue that this large pool of new genes would have enabled the complex regulation required for the development and function of the vertebrate body plan. They integrate these results with expression and pathway data to show that retained ohnologs play important roles in functional categories, such as those required by the nervous system and for locomotion, that are crucial to complex vertebrates.

**Figure 1:**
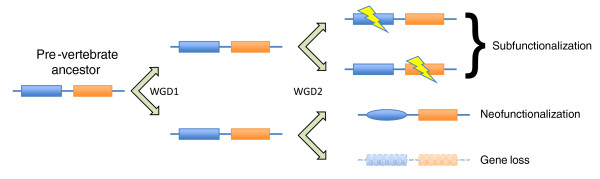
**A simplified schematic diagram of the 2R quadruplication and subsequent gene fates**. A model gene encoding a two-domain protein is duplicated twice. Each of the top two copies loses one domain (function) during subfunctionalization; together they make up the function of the ancestral form. Another copy acquires a new domain and thus a new function (neofunctionalization). The last copy fails to serve a unique function and is lost. A similar process can cause loss and gain of expression regulatory elements.

After WGD, each new ohnolog enters a race to develop an essential function before succumbing to deletion [1]. Some develop variant new functions (neofunctionalization), while other ohnolog pairs reciprocally lose some of their functions or expression pattern (subfunctionalization) (Figure 1). Others survive through gene dosage balance, in which the toxicity of having a double dose of one gene can be offset by the retention of a duplicate of an interacting gene [5]. The relative role of these and more esoteric mechanisms is debated. Multiple mechanisms may act on individual genes: for example, dosage balance may buy time for novel functions to evolve.

The importance of dosage balance is supported by two other findings from this paper. First, small scale duplications (SSDs) that have occurred after 2R show a very different functional bias from that of WGD duplicates: they contain far fewer signaling proteins and transcription factors, but are enriched in immune functions and chromatin modifiers. This suggests that individual duplication of signaling proteins may be toxic or non-functional, requiring the dosage balance of a WGD to survive. A similar bias is also seen in other studies of SSD following WGD, and ohnologs are also underrepresented in copy number variations in human populations, further reflecting their dosage sensitivity [5]. Second, they show that retained ohnologs are more highly connected in pathway and protein interaction maps, further suggesting that they may be required for dosage balance.

The simplest gene dosage models are based on stoichiometric balance between subunits of a stable protein complex. The Huminiecki and Heldin study highlights the limitations of the simple model, since signaling proteins and transcriptional regulators tend to make relatively transient interactions, consistent with their role in information transfer. This suggests that dynamic balancing of signal flux may be as important as structural balances in protein complexes. For instance, duplication of a phosphatase might balance the increased flux from duplication of a corresponding kinase; accordingly, retained ohnologs are specifically enriched for negative regulatory interactions [2]. Dosage balance may also operate in a positive sense: rather than blocking toxicity, the co-duplication of many interacting genes may aid the development of novel pathways and functions.

## Duplicates as an innovation factory

**Figure 2:**
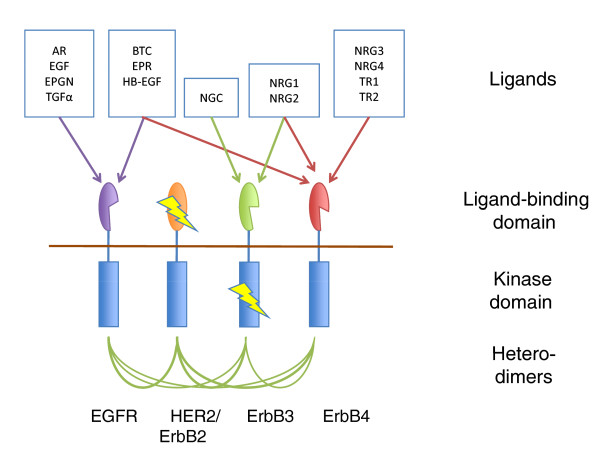
**2R gave rise to a complex EGFR network**. Four EGFR ohnologs have specialized and joint functions: HER2 has apparently lost ligand-binding function, while ErbB3 has almost entirely lost kinase domain function (rectangles, lightning-rod). All six heterodimers can form, with differing signaling capabilities, and duplication and cross-talk between ligands as well as downstream signaling proteins (not shown) further increase the complexity of this system. Abbreviations: AR, amphiregulin; BTC, betacellulin; EGF, epidermal growth factor; EPGN, epigen; EPR, epiregulin; HB-EGF, heparin-binding epidermal growth factor; NGC, neuroglycan-C; NRG, neuregulin; TGFα, transforming growth factor α; TR, tomoregulin.

While dosage balance may explain the initial selective retention of WGD duplicates, development of new functions or expression patterns is the norm in most well studied human gene families. Huminiecki and Heldin observe a divergence of mRNA and protein expression patterns between duplicate genes generated by either WGD or SSD, correlated with age of duplication. In signaling proteins, divergent functions are also common. For instance, all four ohnologs of the epidermal growth factor receptor (EGFR) tyrosine kinase were retained after 2R, giving rise to a complex array of homo- and hetero-dimeric receptors [6] (Figure 2). Subfunctionalization is evident in the almost total loss of catalytic activity in ErbB3 and the apparent loss of ligand binding in ErbB2/HER2, while the concurrent duplications of ligands and downstream signaling genes has further expanded the complexity of this signaling system. EGFRs have proven refractory to SSD in metazoans, and indeed, amplification of the HER2 locus is a major driver for breast cancer, with other EGFR amplicons also reported to be associated with cancer, suggesting that negative selection may be operating on duplication of at least some family members.

Another receptor tyrosine kinase (RTK) family, the Ephs, has expanded by WGD and SSD from one gene in invertebrates to 14 in human, giving rise to a similar explosion in complexity through heterodimerization and ligand cross-talk. This richness is used extensively in developmental patterning, and demonstrates continued evolvability. For instance, in chicken, graded expression of EphA3 across the retina provides the basis for spatial mapping of retinal ganglion cells projecting to the tectum [7]. However, in mouse, EphA3 is not expressed in these cells, and instead EphA5 and EphA6 fulfill this role, suggesting that new and swapped functions can emerge from duplicates long after they have acquired essential roles, and that WGD can represent a quantum leap in the potential for new complexity and evolvability within the vertebrates. We estimate that, excluding the Ephs, 2R caused an expansion of RTKs from 20 to 46, but only two new human RTKs have emerged since then (ES and GM, unpublished): the two rounds of WGD thus seem to have been crucially important in shaping human RTK signaling.

One notable aspect of the patterns reported by Huminiecki and Heldin is how similar they are to those seen in other WGD events [8-10]. Enrichment in signaling proteins and transcription factors has also been seen in WGD from yeast, plants, and fish. Conversely, other genes (mostly those involved in basic cellular processes) preferentially return to singleton status, and similarities in these loss patterns can also be detected across kingdoms. While SSDs show more lineage-specific variability, there are also similarities, such as the increased SSD rate in plant secondary metabolic genes involved in pathogen defense [8] mimicking the increased vertebrate SSD in immune genes.

## 2R: the future

It is tempting to speculate from these observations that WGD produces a consistent drive towards higher complexity [11], and the two rounds of vertebrate WGD doubly so. However, it is a vexed question exactly what is meant by complexity. It is not clear, for example, that fish and frogs, which have undergone an extra round of genome duplication, are more complex than humans, which have not.

The kind of molecular archaeology pursued by Huminiecki and Heldin is not just of academic interest: detailed comparison of ohnologs from many species can provide the unique sequence signatures underlying their specific functions, and patterns of gain or loss can help us to understand functional interactions between genes. As more vertebrate genomes become available, we will gain greater precision in determining orthology, synteny and post-2R changes. Knowing the trends in ohnolog retention and the history of human genes will help us to better understand their dosage sensitivity, and the shared and unique functions of all ohnologs.
